# Immune Response: A Missed Opportunity Between Vitamin D and Radiotherapy

**DOI:** 10.3389/fcell.2021.646981

**Published:** 2021-04-13

**Authors:** Xinyue Yu, Baocai Liu, Ning Zhang, Qian Wang, Guanghui Cheng

**Affiliations:** Department of Radiation Oncology, China–Japan Union Hospital of Jilin University, Changchun, China

**Keywords:** vitamin D, calcitriol, radiation, radiotherapy, immune response, tumor microenvironment

## Abstract

Radiotherapy (RT) is a mainstay treatment in several types of cancer and acts by mediating various forms of cancer cell death, although it is still a large challenge to enhance therapy efficacy. Radiation resistance represents the main cause of cancer progression, therefore, overcoming treatment resistance is now the greatest challenge for clinicians. Increasing evidence indicates that immune response plays a role in reprogramming the radiation-induced tumor microenvironment (TME). Intriguingly, radiation-induced immunosuppression possibly overwhelms the ability of immune system to ablate tumor cells. This induces an immune equilibrium, which, we hypothesize, is an opportunity for radiosensitizers to make actions. Vitamin D has been reported to act in synergistic with RT by potentiating antiproliferative effect induced by therapeutics. Additionally, vitamin D can also regulate the TME and may even lead to immunostimulation by blocking immunosuppression following radiation. Previous reviews have focused on vitamin D metabolism and epidemiological trials, however, the synergistic effect of vitamin D and existing therapies remains unknown. This review summarizes vitamin D mediated radiosensitization, radiation immunity, and vitamin D-regulated TME, which may contribute to more successful vitamin D-adjuvant radiotherapy.

## Introduction

Radiotherapy (RT) is usually the definitive treatment for many types of tumors, including but not limited to colorectal cancer, nasopharyngeal carcinoma, and cervical cancer (Chen et al., 2019; Cohen et al., 2019; Dekker et al., 2019). Resistance to radiation is considered to be an important reason for tumor recurrence and local failure, different cell molecular mechanisms are involved in intrinsic and acquired resistance of cancer cells to therapeutics. Although some strategies, such as radiosensitizers, have been investigated recently, sensitizers have been limited to preclinical studies due to the toxic effects of these agents.

Vitamin D, which is a fat-soluble secosteroid mediating numerous physiological functions (Maurya et al., 2020), has been demonstrated to participate in antitumor activity in many cancers (Keum et al., 2019). Moreover, accumulating data suggest that vitamin D employs several mechanisms to enhance the elimination of irradiated tumor cells (Sundaram and Gewirtz, 1999; Chaudhry et al., 2001; Bristol et al., 2012; Sharma et al., 2014). Thus, a deeper understanding of how vitamin D functions in combination with RT in cancer may help in developing effective sensitizers to overcome radioresistance.

It is worth noting that the immune system plays an important role in the response to RT (Chen et al., 2019). Radiation can lead to both positive and negative regulation of the immune response, and this has been observed not only in tumor cells but also in the tumor microenvironment (TME). Importantly, vitamin D is also involved in the immune microenvironment (Charoenngam and Holick, 2020), although the underlying mechanism has not been clearly elucidated.

Although there is growing awareness of the importance of vitamin D in tumor cell response to radiation, there have been few reviews to report the underlying mechanisms. In this review, we briefly introduce vitamin D and the mechanisms influencing radiosensitivity. Additionally, we discuss how the immune response is regulated in response to RT. Finally, we present the modulation of TME by vitamin D and speculate on the intricate association among vitamin D, radiation, and anti-tumor immunity.

## Vitamin D Metabolism and Epidemiology

Vitamin D is produced from 7-dehydrocholesterol in the human skin after exposure to ultraviolet radiation in sunlight, therefore, it can be influenced by season, latitude, skin pigmentation, and cultural habits. In addition, dietary habits and supplementation can also affect vitamin D levels (Amrein et al., 2020). A two-step catalysis mediated by cytochrome P450 is the crucial process in the production of the steroid hormone calcitriol (biologically active form of vitamin D) (Jones et al., 2014). The less active form of vitamin D, 25(OH)D_3_, is generated after the first hydroxylation by vitamin D 25-hydroxylase (CYP2R1 and CYP27A1) in the liver (Bikle, 2014). 25(OH)D_3_ is found to be the major circulating form of vitamin D in the blood, however, general agreement on the threshold levels has not been defined. Recently, 25(OH)D levels of 75-150 nmol/L (30-60 ng/mL) have been proposed to be the optimal range for vitamin D (Bischoff-Ferrari et al., 2016). The association between 25(OH)D level and cancer risk has been described in several solid cancers (Yao et al., 2017; Ramakrishnan et al., 2019; Yuan et al., 2019), and higher 25(OH)D circulating level contributes to better prognosis in colorectal cancer (Markotic et al., 2019). Subsequently, the kidneys utilize the circulating 25(OH)D_3_ as a substrate and convert it into 1,25-dihydroxy-vitamin D3, which is hydroxylated by CYP27B1 (Jones et al., 2014).

Previous finding supports the role of CYP24A1 in catabolizing 25(OH)D and preventing the formation of 1,25(OH)_2_D_3_ (Jones et al., 2012). Interestingly, either activation of the catabolic enzyme CYP24A1 by calcidol or calcitriol or inactivation of CYP27B1 by calcitriol can lead to a negative feedback loop to regulate the vitamin D level, broadening the role of CYP24A1 as an important mediator of the rate limiting step of not only vitamin D generation but also hormone self-regulation, thus potentially ameliorating hypercalcemia. The hypercalcemia induced by an increased concentration of calcitriol or insufficiency in the blood due to its instability can undoubtedly limit its clinical application. This has eventually led to the exploration of calcitriol analogs that can exert equipotent or increased anticancer actions with less side effects (Jones, 2010).

1,25(OH)_2_D_3_ is able to regulate the expression of several genes depending on the tissues, cell types, and context (Carlberg and Munoz, 2020). By binding to vitamin D receptor (VDR), 1,25(OH)_2_D_3_ facilitates dimerization with the retinoid X receptor (RXR), which fosters nuclear translocation of this complex, and subsequent binding to the vitamin D response elements (VDREs) in the target gene, followed by recruitment of co-modulators. Therefore, calcitriol can interfere with target gene expression in the genomic pathway (Carlberg and Munoz, 2020). It functions in the genomic way by which 1,25(OH)_2_D_3_-VDR-RXR complex is involved, and the non-genomic way, by which a 1,25D-membrane-associated, rapid response steroid-binding protein (1,25D-MARRS) is involved (Hii and Ferrante, 2016).

Several studies have confirmed the ability of vitamin D to affect cell proliferation and differentiation (Feldman et al., 2014; Fernandez-Barral et al., 2020a), and that it has an important role in decreasing the risk of developing multiple cancers (Wu et al., 2019). The association between 1,25(OH)_2_D_3_ and cancer was initially detected in 1981, when inhibition of melanoma cells and differentiation induction of myeloid leukemic cells were reported (Abe et al., 1981; Colston et al., 1981). Since then, anticancer properties of vitamin D have been increasingly confirmed through *in vitro* and *in vivo* studies (Lappe et al., 2017; Jeon and Shin, 2018). Recent research has identified the crucial impact of vitamin D on carcinoma cells, especially in colon and breast cancers (Grant, 2020). In line with the antiproliferative effects of 1,25(OH)_2_D_3_, high VDR expression has also been shown in association with favorable prognosis (Feldman et al., 2014; Carlberg and Munoz, 2020). Although these previous studies have shown the positive role of VDR in patient prognosis, VDR has also been found to be associated with increased cancer risk (Zheng et al., 2017), indicating the controversial role of VDR. Thus, VDR may be a possible prognostic biomarker in patients. Furthermore, several findings support that vitamin D supplementation contributes to favorable prognosis (Ng et al., 2019; Urashima et al., 2019; Yonaga et al., 2019), especially the prognosis improvement compared to those with treatment alone (Wesselink et al., 2020).

## Role of Vitamin D in Radiosensitivity

**FIGURE 1:**
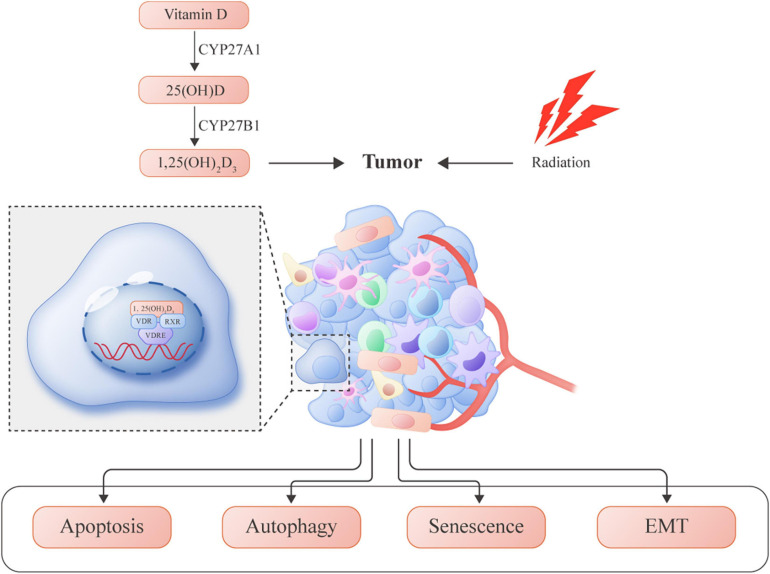
Vitamin D-induced molecular mechanisms involved in enhancing radiosensitivity in several tumors.

More recently, accumulating evidence has confirmed the anticancer role of vitamin D in several cancer models. In addition to the induction of differentiation and proliferation inhibition, the role of vitamin D as a magnifier of radiation response is emerging. Evaluation of combined therapy was performed in preclinical studies, showing synergistic or additive antitumor effectiveness. To date, the molecular mechanisms by which vitamin D potentiates the antitumor effects of RT are only partially known, and need further clarification. The antitumor actions of vitamin D are carried out through several mechanisms, such as induction of apoptosis, inhibition of proliferation, and suppression of angiogenesis. Additionally, vitamin D can also potentiate the antitumor effects of RT through different pathways. A summary of previous literature on the role of vitamin D to enhance radiation sensitization in cancer is presented in Figure 1.

### Apoptosis

Cancer relapse occurs through multiple mechanisms, most of which are mediated by insufficient apoptosis. Increased DNA fragmentation induced by additive EB1089 in MDA-MB-231 cells was associated with increased responsiveness to radiation (Sundaram and Gewirtz, 1999). Although the number of apoptotic cells triggered by radiation alone appeared to be minimal in MCF-7 cells, the rate and extent of cytotoxicity in irradiated cells were enhanced when combined with ILX-23-7553 (vitamin D analog) (Chaudhry et al., 2001). It is important to emphasize that vitamin D3 and EB1089 promote the inactivation of BCL-2 (Simboli-Campbell et al., 1997), which is an anti-apoptotic protein. Additionally, high radiation doses have been correlated with more adverse events. In prostate cancer, vitamin D3 achieved equal therapeutic efficacy by inducing apoptosis along with marked attenuation of the radiation dose, thereby mitigating the side effects associated with a high radiation dose (Dunlap et al., 2003). In preclinical studies, these high-dose strategies show a weaker relationship with clinical RT, necessitating an experimental fractionation dose. The combination of ILX-23-7553 with fractionated radiation (5 × 2Gy, 3 days) demonstrated an advantage in inducing the apoptosis of MCF-7 cells; conversely, it appeared to have no impact on normal human fibroblasts, thus supporting the tumor-specific role of ILX-23-7553 (Polar et al., 2003). Similarly, a relationship between fractionated radiation and EB1089 has been reported in breast cancer: combination treatment led to a higher apoptotic rate than that with radiation alone and showed no detectable toxicity in normal breast epithelial cells or BJ fibroblast cells (DeMasters et al., 2004).

Moreover, it is known that radiation can directly induce cell death by DNA damage or indirectly by production of reactive oxygen species (ROS). When intrinsic resistance develops in tumor cells, ROS clearance is enhanced to ameliorate the oxidative stress. Xu et al. (2007) showed that RelB triggered by radiation resulted in the protection of irradiated cells, whereas vitamin D3 ablated this protection. As a member of the NF-κB family, RelB can be inactivated by VDR-mediated transcriptional repression. Similarly, in breast cancer cells pre-treated with vitamin D3, sensitivity to radiation was increased accompanied with down-regulation of RelB (Mineva et al., 2009), again indicating that RelB was a target gene regulated by vitamin D in response to radiation.

### Autophagy

As a source of cellular stress, radiation can also induce autophagy, which is a homeostasis mechanism for cellular stress and is mediated by a series of autophagy-related proteins. This process could also be influenced by vitamin D. Gewirtz (2014) first summarized the four faces of autophagy, which are the different types of autophagy that may play crucial roles in response to conventional therapies: (i) cytotoxic autophagy, (ii) cytoprotective autophagy, (iii) cytostatic autophagy, and (iv) non-protective autophagy.

#### Cytotoxic Autophagy

Demasters et al. (2006) indicated that autophagic cell death could be an important tumor cell elimination mechanism for combinatorial EB1089 and radiation in breast cancer. Cytotoxic autophagy is characterized by enhanced cell death, which is accompanied with earlier occurrence and greater extent of autophagosome, but no other apparent cell death type.

#### Cytoprotective Autophagy

Response to a single or combination treatment is not simply the result of a uniform type of autophagy, there is evidence that cytoprotective autophagy induced by radiation alone may be converted to cytotoxic autophagy when combined with vitamin D3 (Wilson et al., 2011). This further supports the view that dual functions of autophagy may be exhibited concomitantly. The cytoprotective form of autophagy is often considered as a mechanism of drug and radiation resistance in tumors (Ko et al., 2014). Consequently, autophagy inhibitors have been proposed to counteract the elevated cytoprotective autophagy induced by RT to improve radiosensitivity. Several studies have indicated that vitamin D3 shifted the autophagy type from cytoprotective to cytotoxic, which has been considered an autophagic switch (Wilson et al., 2011; Bristol et al., 2012).

#### Cytostatic Autophagy

Both cytotoxic and cytoprotective autophagy are closely related to the alteration of the autophagy flux level, whereas a cytostatic form of autophagy has been shown to exert beneficial antitumor effects on non-small cell lung carcinoma independent of the autophagy flux alteration. In a study by Sharma et al. (2014), EB1089 was revealed to increase radiation sensitization by inducing a growth-arrest status with no autophagy alteration or direct cell killing; interestingly, pharmacological inhibition or genetic silencing of autophagy rescued the tumor cells from the cytostatic status. EB1089 is possibly effective in switching the autophagy to cytostatic mode which appears to be involved in growth arrest. Therefore, autophagy primarily induced by radiation can be maintained, whereas the nature can be shifted to antitumor activity.

#### Non-protective Autophagy

Unlike cytoprotective autophagy, where autophagy inhibition results in an enhanced response to treatment, or cytotoxic and cytostatic autophagy where autophagy inhibition leads to reduced therapy efficiency, inhibition of non-protective autophagy shows no association with response to therapeutics. A study by Chakradeo et al. (2015) confirmed this finding by investigating whether cytoprotective autophagy induced by radiation in multiple cell lines was blocked by pharmacological inhibition or genetic silencing of autophagy genes. They found that inhibition of autophagy failed to influence the radiation sensitivity of p53 null cells, which seems to support the mysterious non-protective form of autophagy.

### Senescence

In a previous study, EB1089 was found to enhance cell apoptosis in response to radiation; the study also claimed that EB1089 had no perceptible effect on preventing senescence but only delayed the emergence of senescence (DeMasters et al., 2004). Similar studies on vitamin D3 mediated radiosensitivity also mentioned the occurrence of cell senescence (Demasters et al., 2006; Wilson et al., 2011).

#### Therapy-Induced Senescence

Traditionally, senescence was considered irreversible; however, recently, the focus has been on the capability to regain proliferation instead of quiescence in response to radiation. TIS was an accelerated form of senescence (or premature), different from the replicative senescence in aging cells. There is increasing evidence that low-dose radiation interferes with TIS (Yu et al., 2018). Under this condition, senescent cells can escape from direct damage due to RT and enter temporary dormancy; once re-activated, the surviving cells re-emerge from the dormant state and develop into a more aggressive phenotype (Rodier and Campisi, 2011). This is also termed “pseudo-senescence” or “senescence-like arrest.” Some clinical reports have demonstrated that patient prognosis was negatively correlated with the expression of senescence markers when exposed to radiation (Fischer et al., 2011).

#### Senescence-Associated Secretory Phenotype

Radiation influences not only irradiated cells but also the TME, or the so called SASP (Faget et al., 2019). SASP can influence the neighboring non-irradiated cells by releasing a series of pro-inflammatory chemokines and cytokines such as IL-1β, IL-6, and CXCL1 into the surrounding environment (Acosta et al., 2013). Such molecules can hinder the success of RT. This bystander effect induced by radiation might be the mechanism by which a tumor treated with primary therapy becomes refractory to further treatment. This premise has been supported by Huang et al. (2014), who established a bystander model by treating non-irradiated cells with a conditioned medium acquired from irradiated senescent MDA-MB-231-2A cells. They demonstrated that the conditioned medium could lead to the invasion and migration of neighboring cells mediated by the JAK2-dependent AKT and STAT3 pathways. This non-targeted effect of radiation requires potential therapeutics for a better regulation between the target site and the surrounding microenvironment.

#### Vitamin D and Senescence

Studies on the elimination of TIS are underway for the prevention of disease relapse (Gorgoulis et al., 2019; Short et al., 2019). Notably, VDRE is a promoter of the gene encoding p21; thus, vitamin D3 could directly regulate p21 by binding to VDRE (Ylikomi et al., 2002). Elevated levels of IL-6 and IL-8 are associated with paracrine secretion in the SASP phenotype, and vitamin D3 has been proven to exert anti-inflammatory effects in prostate cancer through the inhibition of IL-6, IL-8, and TNF-α (Giangreco et al., 2015). Mechanistically, vitamin D was shown to inhibit the IL-6 production through the inactivation of p38MAPK (Nonn et al., 2006). These observations imply that vitamin D3 may be a potential senolytic that can eliminate senescence-related effects, thereby enhancing sensitization to radiation.

### Epithelial-to-Mesenchymal Transition

EMT is a reversible process, which usually involves an initial loss of the differentiated phenotype to the migratory phenotype as circulating form in the bloodstream, and subsequent mesenchymal–epithelial transition (MET) for initial colonization leading to metastatic niches, thus generating intratumoral phenotypic heterogeneity (Angela Nieto, 2017). Usually, EMT is accompanied with diminished apoptosis and increased stemness, and both effects are linked to resistance to conventional therapies (Dongre and Weinberg, 2019). Data on colorectal cancer have shown that calcitriol significantly enhanced the therapeutic effects of radiation regulated by EMT (Findlay et al., 2014). In this study, Slug was involved, and overexpression of Slug in calcitriol-sensitive cell lines abrogated the radiosensitization effect. DNA damage repair is a major regulator of treatment response and also involved in radioresistance. ZEB1 was reported to promote DNA damage repair (Zhang et al., 2014). Besides, the presence of Snail was correlated with decreased apoptosis mediated by p53 (Kurrey et al., 2009). Furthermore, upregulation of Slug by IR reversely contributed to inhibition of PUMA, thus decreasing apoptosis (Wu et al., 2005). These data establish EMT as a sensitization switch which regulates the treatment response, and harness of EMT related transcriptional factors may be a potential strategy for enhancing response to RT. Additionally, there is evidence that calcitriol can directly influence tumor-initiating cells (TICs, also known as cancer stem cells), which demonstrated that calcitriol in combination with radiation could inhibit spheroid formation more than either treatment alone; furthermore, this effect could be abolished by the overexpression of β-catenin (Jeong et al., 2015). It has been postulated that non-cancer stem cells are more sensitive to treatment, and EMT can directly characterize epithelial cells of the stem-cell properties, therefore, understanding the impact of calcitriol on EMT and CSCs might provide a novel insight into its effect on radiosensitivity.

Nevertheless, the scant molecular data have revealed that vitamin D may enhance the response to radiation at different levels. The reported molecular mechanisms involve the potentiation of existing apoptosis and the inhibition of protective autophagy. Moreover, the role of vitamin D in senescence and EMT transition requires further investigation. The available evidence strongly suggests that 1,25(OH)_2_D_3_ could be considered for combination therapy for cancer.

## Radiation Immunity

**FIGURE 2:**
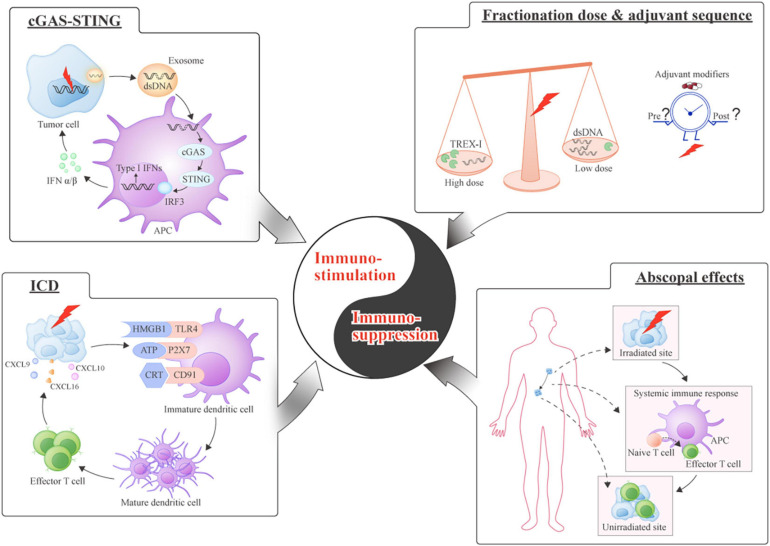
Equilibrium between immuno-stimulation and immuno-suppression induced by radiation. Immuno-stimulation (Left): (cGAS-STING) cGAS-STING-mediated type I interferons (IFNs) are released by sensing double-stranded DNA (dsDNA) and contribute to the immune response; (Immunogenic cell death [ICD]) A schematic of the series of molecules binding to their receptors during immunogenic cell death. Immuno-suppression (Right): (Fractionation dose and sequence) The dose of RT influences immunomodulation through the production of Three-Prime Repair Exonuclease 1 (TREX1); the introduction of immune modifiers should be optimally timed; (Abscopal effects): The priming of antitumor immunity by local RT causes the distant tumor sites to shrink by activating systemic immune response.

As summarized above, vitamin D demonstrates a synergistic effect with radiation through various mechanisms. However, given the complexity of the direct impact of radiation on tumor cells and the indirect impact on the TME, it is worth noting the role that immune response plays in the response to RT. It has been traditionally thought that RT is an approach to suppress the immune system when harnessed for allogeneic transplantation (Rodier and Campisi, 2011). Recently, reactivation of the immune system, or the 6th R of radiobiology, has been proclaimed as the emerging target for RT (Fischer et al., 2011). RT participates in numerous steps of the immunological process (Figure 2). Therefore, the ultimate impact of vitamin D may be dependent, in part, on the radiation-induced TME, and the role that the immune system plays in the overall TME.

### RT Induced Immuno-Stimulation

#### Immunogenic Cell Death

Evidence shows that ICD is the dominant process responsible for the higher therapeutic efficacy of concurrent chemoradiotherapy than single chemotherapy (Formenti and Demaria, 2008). The effects of RT are far beyond tumor size reduction; RT converts the irradiated site into an immunogenic hub by releasing damage associated molecular patterns (DAMPs), the so-called “*in situ* vaccine” that contributes to the priming of the systemic immune response (Carvalho and Villar, 2018). Increasingly, evidence indicates that radiation-induced DAMPs can exert adjuvanticity by converting the “immune-cold” tumor into the “immune-hot” tumor (Whiteside et al., 2016). “Cold” tumor normally does not respond to conventional therapies. This conversion relies on the ability of RT to induce primary tumor to be the immunogenic hub, thus significantly improving the effector T cell response (Zheng et al., 2016). A better appreciation of the intricate interaction between immune cold-to-hot conversion and radiotherapy is emerging (Galon and Bruni, 2019), which could shed light on tumors respond poorly to existing treatments. The mechanism of ICD includes CRT translocation to the dendritic cell (DC) surface promoting antigen presentation, release of HMGB1 from the dying cells to activate the Toll-like receptor 4 (TLR4) pathway, and ATP binding to the P2X7 receptor in DCs (Apetoh et al., 2007; Obeid et al., 2007; Ghiringhelli et al., 2009). Radiation can potentiate ICD through any of these three steps (Golden et al., 2014). Various DAMPs can be induced by radiation. HMGB1, as one of the DAMPs, has been shown to boost antigen presentation by DCs; other factors such as TLR4, which contributes to the binding of HMGB1 to DCs, are also triggered in response to radiation (Apetoh et al., 2007).

#### Antigenicity

Tumors with a higher antigen load have a greater tendency to induce the activation of naive T cells by DCs, leading to the possibility of low-immunogenic tumors inducing an immune response (Galluzzi et al., 2017). Following the release of tumor associated antigens (TAA) such as DAMPs, tumor-specific T cells are trafficked back to the tumor site, and the radiation-triggered immune response can therefore be amplified (individualized vaccination) (Vanpouille-Box et al., 2017). Radiation has been proven to lead to the upregulation of MHC-I, which further enhances the efficacy of antigen presentation (Reits et al., 2006). A similar effect has been observed in DCs, manifesting as improved maturation and recruitment of DCs to the irradiated site. The T cell function in response to RT was first described by Stone et al. (1979), who demonstrated that the attenuation of the radiation efficacy correlated with immune insufficiency. Experimental data have shown that the antitumor immune effects of RT could be attributed to CD8+ T cell infiltration (Lee et al., 2009).

#### Chemokines and Cytokines

Specific chemokines and chemokine receptors are crucial for T-cell trafficking to the tumor site. For instance, irradiated tumors secrete C-X-C motif ligand 9 (CXCL9), CXCL10, and CXCL16, which bind to their receptors C-X-C chemokine receptor type 3 (CXCR3) or CXCR6 expressed on T cells or T helper 1 cells (T_H_1), and can facilitate the homing of CD8 T-cells to the irradiated site (McLaughlin et al., 2020). DNA damage has recently been identified to play a novel role in anti-tumor immunity induced by RT (Harding et al., 2017; Mackenzie et al., 2017), and the presence of double-stranded DNA (dsDNA), a recognized type I interferon (IFN-I) initiator, has been shown to elicit a tumor-specific T cell response (Deng et al., 2014; Vanpouille-Box et al., 2017). The cGAS-STING (cyclic GMP-AMP synthase-stimulator of interferon genes) pathway is of great significance as it is involved in dsDNA sensing and production of IFN-I during the radiation response (Deng et al., 2014; Harding et al., 2017; Mackenzie et al., 2017). The micronuclei derived from the damaged DNA can be transported by the nucleic acid sensors, cyclic GMP-AMP (cGAMP), to the STING dependent pathway and promote IFN-I production. Three-Prime Repair Exonuclease 1 (TREX1), upstream of cGAS, is a known exonuclease that can be transferred by exosome and has been shown to be associated with the degradation of dsDNA in irradiated cells (Diamond et al., 2018). Exosome is a particular form of extracellular vesicles with a size range of 40 to 160 nm in diameter and carries different types of cargoes inside (Kalluri and LeBleu, 2020). Previous evidence indicates that radioresistance is correlated with tumor derived exosomes (Ni et al., 2019). The most straightforward reply for how exosome respond to radiation is the alteration of its content because of altered TME induced by radiation (Diamond et al., 2018). The production of TREX1 is radiation dose-dependent and may lead to immune failure when receiving RT at a dose more than 12 Gy (Vanpouille-Box et al., 2017). Therefore, the commonly seen therapeutic resistance in response to high-dose RT may result from concentration-dependent TREX1, which can modulate cytosolic dsDNA and thus influence the immune response. It is important to understand that the tumor-cell-intrinsic sensing remains unclear. There is evidence that caspase 9 (CASP9) signaling hijacked by the irradiated tumor cells can result in acquired resistance to radiation by the inhibition of innate DNA sensing (Han et al., 2020). When CASP9 was blocked using a pan-caspase inhibitor, a thousand-fold increase in IFN-I appeared in response to RT. Caspase appears to be involved in the immune response to radiation mediated by the innate DNA sensor.

### RT Induced Immuno-Suppression

#### Immuno-Suppression

Since cancer is commonly based on the equilibrium between pro-immune and anti-immune effects in a degree sufficient to cause substantial cell death, T cells can induce successful immunization for achieving tumor elimination. An inhibitory TME often acts as a signal for immunosuppression, and this aberrant milieu influences the intrinsic properties of the surrounding cells. Specific immune suppressive cytokines are important for this milieu. Transforming growth factor-β (TGF-β), which can be induced by radiation, has been shown to impair antigen-presentation by DCs and can impede effector T cell differentiation (Tauriello et al., 2018). A study has indicated that T cell-mediated tumor rejection was acquired only when combined with anti-TGF-β (Vanpouille-Box et al., 2015). In addition, the remarkable myeloid-derived suppressor cells (MDSCs) induced by RT can also lead to immunosuppression mediated by TGF-β (Vatner and Formenti, 2015). Meanwhile, up-regulation of MDSCs in response to radiation is also associated with increased anti-programmed death-ligand 1 (PD-L1) expression on the cell surface of MDSCs (Dovedi et al., 2017). MDSCs can differentiate into mature macrophages, and there is evidence that radiation can cause the macrophage polarization into the M2 phenotype, thus attenuating the response to therapy (Tsai et al., 2007). Moreover, suppressive chemokines such as C-C motif chemokine 2 (CCL2) or CCL5 released from irradiated cells can recruit MDSCs and regulatory CD4 T cells (Tregs) to the tumor site (Connolly et al., 2016). Intriguingly, although IFN-I derived from the cGAS-STING pathway plays an important role in the antitumor immune response induced by RT, long-term chronic interferon-driven basal interferon-stimulated gene (ISG) was also correlated with T cell dysfunction (McLaughlin et al., 2020). Thus, the dual target roles of IFN-I need further investigation for effectively mitigating immunosuppression.

#### Abscopal Effect

The ability of RT to inhibit tumor growth far from the irradiated site is called the abscopal effect (Formenti and Demaria, 2009). The link between the abscopal effect and systemic immunity was first reported by Demaria et al. (2004), who claimed that the antitumor immune response triggered by radiation can also elicit effective eradication of the non-irradiated tumor site. As an immunogenic hub, the field directly exposed to radiation sustains the *in situ* vaccine effect; however, this may be insufficient. The possible theories regarding how radiation can trigger the abscopal effect are based on the equilibrium between the immunostimulatory and immunosuppressive effects. As explained above, radiation not only has an immunostimulatory effect on the irradiated site but also promotes an immunosuppressive response in the surrounding environment. In a preclinical experiment, the abscopal effect was observed under blockade of immunosuppression (Levy et al., 2016), which supports the potential use of immune modifiers for this effect. Logically, TAAs play the role of mediators in the abscopal effect by shuttling outside the radiation field; however, when administered alone, radiation or immune checkpoint inhibitors (ICIs) did not inhibit growth in all the metastatic niches, suggesting that the antigenic overlap between the irradiated and non-irradiated sites was required to elicit an abscopal effect (Formenti et al., 2018). Activation of systemic antitumor immunity undergoes numerous processes including neoantigens releasing and priming of T cell infiltration. Notably, the abscopal effect is previously rare and single-site irradiation only shows some modest success, does not substantially increase the response rate (Luke et al., 2018). Therefore, we should rethink the importance of tumor heterogeneity. Radiation to multiple sites has been suggested to surmount this barrier and lead to optimal effectiveness of RT, which can be a meaningful strategy to prime systemic immune response (Brooks and Chang, 2019).

#### Radiation Fractionation and Sequence

Of note, the promotion or inhibition of the immune response triggered by radiation depends on various factors, such as the fractionation dose. A radiation dose of more than 7.5 Gy but not 5 Gy was revealed to stimulate the systemic immune response in a low immunogenic tumor (Stone et al., 1979), and a mathematical model appeared to allow the maximum immunity level in a range of 10-13 Gy (Lee et al., 2009). Conventional dose fractionation or single high-dose RT increases the amount of MDSCs, conversely, this can be reverted by hypofractionation (McLaughlin et al., 2020). There is evidence that 8 Gy × 3 fractions or 6 Gy × 5 fractions, but not a single high dose, can activate the immune response more intensively (Apetoh et al., 2007). Furthermore, a single dose of 20 Gy did not have a synergistic effect with additive immune modifiers. TREX1 induced by RT might help to elucidate the abrogation of the immune response (Nonn et al., 2006). Commonly, antitumor immune response with radiation alone has a limited effect, and optimal stimulation of the adaptive immunity requires the aid of ICIs. Recently, immune checkpoints, such as the programmed cell death protein 1 (PD-1) or cytotoxic T lymphocyte-associated protein 4 (CTLA-4), as co-inhibitory receptors on T lymphocytes, have been selected as targets to reactivate T cell function (Harding et al., 2017). This application of ICIs can make the paradigm shift to RT and vice versa. Additionally, the increased antitumor activity also depends on whether the immune modifiers are administered, before, after, or concurrently with RT. This might be due to the functional mechanisms of modifiers to inhibit immunosuppression. For example, therapeutic benefit could be acquired only when anti-PD-L1 was applied concomitantly with RT (Mackenzie et al., 2017). It seems that the effectiveness of anti-PD-L1 therapy depends on the upregulation of PD-L1 on the cell surface, which should be first activated by radiation.

A likely explanation for the mixed results is that a specific therapeutic window is needed for RT to remove the immune barriers when acting synergistically with immune adjuvants. Additional evidence on how conventional fractionation or hypofractionation influences the immune system should be acquired for harnessing the benefits of combination treatment. There is a very delicate balance between the immunostimulation and immunosuppression induced by RT; consequently, effective cancer treatment is determined by an optimal fractionation scheme combined with specific immune modifiers along with suitable timing.

## Vitamin D and Immunity

Although the crucial role of the TME during RT is widely accepted, studies on how the radiation-induced TME can be reprogrammed by adjuvants are scarce. Recent study has demonstrated that vitamin D can also modulate tumor stromal cells (Sherman et al., 2014), and the excellent review has summarized the effects of vitamin D on the TME (Wu et al., 2019). The primary association between vitamin D and immunity is mainly thought to be based on energy metabolism and defense against infections. However, in recent years, vitamin D has been shown to be a multifaceted regulator of the immune system (Hanel and Carlberg, 2020). Vitamin D has been used for the treatment of autoimmune disorders by attenuation of the inflammatory immune response (Dimitrov et al., 2017), and it has also been shown to benefit organ transplant patients by inhibiting autoimmunity (Zhou et al., 2017). In fact, although vitamin D induces partial immunosuppression in normal tissues, the long-term effect of chronic inflammation control prevents tumorigenesis, allowing for the antitumor immunity induced by vitamin D.

Study has shown that the immune system can also affect vitamin D production (Liu et al., 2006). A high level of 25(OH)D_3_ in plasma led to a lower risk in the colorectal cancer subtype with an intense immune reaction, but had no effect on low degree reaction subtypes (Song et al., 2016). This further supports that sufficient immunity is necessary for vitamin D to exert its antitumor effect. Apart from kidney tubular cells, immune cells also express CYP27B1 and VDR, which reinforces the important role of vitamin D in regulating immune functions (Wei and Christakos, 2015; Christakos et al., 2016). Many types of immune cells such as DCs, CD4, and CD8 T cells expressing VDR (Lu et al., 2018) and CYP27B1 can produce the active metabolite 1,25(OH)_2_D_3_, which can maintain a healthy immune system (Barragan et al., 2015).

### Inflammation

Not only the infiltrating immune cells but also cytokines and chemokines in the TME usually influence the tumor response to treatment (Diakos et al., 2014). Vitamin D regulates the inflammatory microenvironment through several mechanisms (Liu et al., 2018). NF-κB plays an important role in regulating immune response (Miraghazadeh and Cook, 2018), and evidence supports the role of VDR antagonist in suppressing p65 activation (Tse et al., 2010). It was also found that vitamin D increased the infiltration of CD8+ T cells, and this was due to the suppression of IL-6 in the TME (Karkeni et al., 2019). Moreover, 1,25(OH)_2_D_3_ was effective in suppressing IL-8, which was based on the inhibition of NF-κB activation (Yang et al., 2018).

### Cancer-Associated Fibroblasts

CAFs are a heterogeneous population of cells in the TME, derived from tumor cells or tumor stroma cells, and are usually involved in tumor progression and therapeutic resistance (Augsten, 2014). Therefore, a strategy to target the CAFs is necessary (Chen and Song, 2019). Recent data indicate that calcipotriol (VDR agonist) can enhance the therapeutic efficacy by reducing inflammation and fibrosis in pancreatic cancer (Sherman et al., 2014). In line with these data, analyses of patients have reported that high VDR expression in CAFs is associated with better prognosis (Ferrer-Mayorga et al., 2017). These findings are clinically relevant, which indicates that VDR agonists can exert antitumor actions on tumor stromal cells and patients in carcinoma VDR-negative status may still benefit from vitamin D treatment. In recent years, recognition of the crucial role played by exosome in cancer has led to the novel insight for selectively targeting cancer cells (Knox et al., 2020). In this context, a study (Kong et al., 2019) reported that vitamin D decreased the amount of exosomes secreted by the CAFs and thus inhibited the tumor promoter miR-10a-5p in pancreatic cancer.

### Cancer Stem Cells

CSCs are a subpopulation of cells characterized by self-renewal due to the accumulation of genetic and epigenetic alterations, and possibly make an important contribution to therapeutic resistance. Moreover, inhibition of CSCs by vitamin D has been described in prostate and breast malignancies as a promising treatment strategy (So and Suh, 2015). 1,25(OH)_2_D_3_ has been found to reduce sphere formation in breast cancer, with downregulation of stem cell markers and NOTCH pathway genes (Shan et al., 2017). Organoids have been proposed to represent the *in vivo* situation, as three-dimensional structures generated by primary normal or cancer stem cells isolated from the patients. On the one hand, 1,25(OH)_2_D_3_ can induce cell differentiation in colon tumor organoids and lead to a more epithelial phenotype (Fernandez-Barral et al., 2020b). On the other hand, 1,25(OH)_2_D_3_ upregulates stemness-related genes and downregulates differentiation genes in normal rectum organoids (Costales-Carrera et al., 2020). These results demonstrate the different roles of vitamin D in normal stem cells and colon CSCs. Recently, it has been demonstrated that vitamin D induced significant downregulation of stemness-related genes compared to imatinib alone, indicating the apparent amplified function of vitamin D on CSCs (Kotlarz et al., 2019). Likewise, reduction of MCF-7 stem cell subpopulation can be induced by VDR overexpression, which elevates sensitivity to tamoxifen (Zheng et al., 2018).

The above data clearly demonstrate that 1,25(OH)_2_D_3_ can suppress tumor progression by modulating the TME. Initially, it has been shown that immune cells and tumor stromal cells express VDR, which contributes to 1,25(OH)_2_D_3_ responsiveness. Supporting this, vitamin D correlates inversely with the CAFs in the surrounding TME. In addition, the inhibition of CSCs is probably also a consequence of TME regulation by 1,25(OH)_2_D_3_. Similar functional effects were observed on the novel three-dimensional structures of organoids. Taken together, vitamin D modulates the TME in diverse ways, which strongly indicates the multi-level anticancer actions of vitamin D in various cancers.

## Future Perspectives

This review aimed to bring together two different fields, namely, vitamin D and radiation, which have rarely been linked before. We used the TME as the bridge between the two fields. However, further investigation is required before we can fully elucidate the impact of vitamin D on radiation. Notable evidence reported in previous studies has highlighted the importance of the TME in the treatment response of cancer. Molecular scenarios induced by radiation in cancer also demonstrate the remarkable functions of the TME. We hypothesize that, if the immunosuppression caused by radiation can be weakened or subtracted by vitamin D, the equilibrium will be broken and immunostimulation will be in dominancy. Supporting this, suitable vitamin D intervention in combination with radiation can induce an antiproliferative additive effect, and this effect of RT may be derived from not only directly causing cancer cell death but also indirectly reprogramming the TME. This may widen the perspective on vitamin D with regard to its immune modulatory role, which is essential for the treatment of autoimmune disorders. Overall, radiation therapy is complex with the involvement of intricate immune modulation and multiple types of cancer cell death. Additional research is needed to elucidate the underlying mechanisms and the potential utility of vitamin D in RT.

Nonetheless, more investigations are needed to confirm whether there is existing resistance to vitamin D itself, accompanied with the detection of a vitamin D response-dependent biomarker, which could facilitate the selection of patients with a higher likelihood of response to vitamin D, and decide the biologically optimal dose of vitamin D for achieving maximum health benefit. Is there a scheme to satisfy the target doses by controlling the local concentration of calcitriol? For greater benefits, the development of VDR agonists is recommended, which is deemed to acquire the equi-effective but less hypercalcaemia effect. The timing of vitamin D initiation during combination therapy is another important issue. For best regimen, whether the agent should be supplemented continuously or not? Does radiation alter vitamin D metabolism indirectly? These explorations may contribute to the discovery of potential cost-effective and efficient agent for combination treatment with conventional therapeutics.

## Author Contributions

GC conceptualized and supervised the conception of the manuscript. GC and XY coordinated and performed the literature search with BL, NZ, and QW contribution. XY and GC designed and performed figures, and wrote the manuscript with important inputs from all authors. All authors reviewed and agreed with the content of the manuscript.

## Conflict of Interest

The authors declare that the research was conducted in the absence of any commercial or financial relationships that could be construed as a potential conflict of interest.
